# Aqueous Extract of *Annona macroprophyllata*: A Potential **α**-Glucosidase Inhibitor

**DOI:** 10.1155/2013/591313

**Published:** 2013-11-04

**Authors:** F. Brindis, M. E. González-Trujano, M. González-Andrade, E. Aguirre-Hernández, R. Villalobos-Molina

**Affiliations:** ^1^Unidad de Biomedicina, Facultad de Estudios Superiores-Iztacala, Universidad Nacional Autónoma de México, Avenida de los Barrios 1, Los Reyes Iztacala, 54090 Tlalnepantla, MEX, Mexico; ^2^Laboratorio de Neurofarmacología de Productos Naturales de la Dirección de Investigaciones en Neurociencias, Instituto Nacional de Psiquiatría Ramón de la Fuente Muñiz, Calzada México-Xochimilco 101, Colonia San Lorenzo Huipulco, 14370 México City, DF, Mexico; ^3^Instituto Nacional de Medicina Genómica (INMEGEN), Periférico sur 4809, Arenal Tepepan, Tlalpan, Secretaría de Salud, 14610 México City, DF, Mexico; ^4^Departamento de Ecología y Recursos Naturales Facultad de Ciencias, Universidad Nacional Autónoma de México, Ciudad Universitaria Coyoacán, 04510 México City, DF, Mexico

## Abstract

Annona genus contains plants used in folk medicine for the treatment of diabetes. In the present study, an aqueous extract prepared from *Annona macroprophyllata* (Annonaceae, also known as *A. diversifolia*) leaves was evaluated on both the activity of yeast **α**-glucosidase (an *in vitro* assay) and sucrose tolerance in Wistar rats. The results have shown that the aqueous extract from *A. macroprophyllata* inhibits the yeast **α**-glucosidase with an IC_50_ = 1.18 mg/mL, in a competitive manner with a *K*_*i*_ = 0.97 mg/mL, a similar value to that of acarbose (*K*_*i*_ = 0.79 mg/mL). The inhibitory activity of *A. macroprophyllata* was reinforced by its antihyperglycemic effect, at doses of 100, 300, and 500 mg/kg in rats. Chromatographic analysis identified the flavonoids rutin and isoquercitrin in the most polar fractions of *A. macroprophyllata* crude extract, suggesting that these flavonoids are part of the active constituents in the plant. Our results support the use of *A. macroprophyllata* in Mexican folk medicine to control postprandial glycemia in people with diabetes mellitus, involving active constituents of flavonoid nature.

## 1. Introduction

Diabetes mellitus (DM) is a chronic disease associated with abnormal and constant high blood glucose (hyperglycemia) that results from defects in insulin secretion, action, or both. In type 2 diabetes mellitus (DM2), postprandial hyperglycemia is important in the development of the disease; it is associated with micro- and macrovascular complications, and it has been proposed as an independent risk factor for cardiovascular disease [1, 2]. The postprandial phase is characterized by a rapid and increased glycemia. These postprandial “hyperglycemic spikes” may be relevant to the pathophysiological conditions of late diabetes [3, 4].

One important factor that arises in postprandial hyperglycemia is the fast uptake of glucose in the intestine, in which *α*-glucosidases hydrolyze starch and oligosaccharides [5]. The *α*-glucosidases, exoglycosidases, found in the luminal surface of enterocytes contain maltase/glucoamylase and sucrose/isomaltase activities [6]. It is believed that inhibition of these enzymes can effectively control the postprandial elevation of blood glucose. Therefore, an important strategy for managing postprandial hyperglycemia is to inhibit *α*-glucosidase activity [7]. The *α*-glucosidase inhibitors, such as acarbose, have been used in the clinic to control blood glucose increase, especially postprandial, in DM2 [8]; these chemical drugs have strong inhibitory activity against *α*-glucosidases but have the disadvantage of undesirable side effects such as abdominal distention, flatulence, meteorism, and diarrhea [9]. Previous research indicates that side effects could be caused by excessive inhibition of pancreatic *α*-amylase, resulting in the abnormal bacterial fermentation of undigested carbohydrates in the colon. Therefore, more effective inhibitors for the enzymes should have strong inhibitory effect against *α*-glucosidase and mild inhibitory effect against *α*-amylase, which can be an effective therapy for managing postprandial hyperglycemia with minimal side effects [10].

Annonaceae is a family of plants consisting of 2300 to 2500 species included in more than 130 genera; in fact, it is the largest family of the order Magnoliales. Only four genera (*Annona, Rollinia, Uvaria,* and *Asimina*) produce edible fruits such as annona [11–13]. *Annona macroprophyllata *Donn. Sm. is a species belonging to the *Annona* genus; it is a common tree in central México known as “ilama” (States of Colima, Guerrero, and México); whereas in the southeast (Tehuantepec region and Yucatán) it is called “papauce” or “anona blanca” [14, 15]. The fruits of this plant are used as food, but its leaves are employed as anticonvulsant [16], as well as analgesic and anti-inflammatory agents in traditional Mexican medicine [17]. However, so far there are no scientific reports supporting its probable antihyperglycemic properties, even though other species of the genus are known to have those properties, that is, *Annona squamosa, A. muricata*, *A. glabra,* and *A. cherimola *[18–20]; then we have used a chemotaxonomic criterion in order that *A. macroprophyllata*, besides its described medicinal properties [14–17], was investigated to search for its likely antidiabetic properties.

## 2. Materials and Methods

### 2.1. Plant Material and Extract

*Annona macroprophyllata *Donn. Sm. specimens were collected in Tejupilco, Guerrero in September 2010. Dr. E. Cedillo-Portugal, botanist from the Universidad Autónoma de Chapingo (UACH), certified the authenticity of the plant, and a voucher specimen (AN9702) was deposited in the herbarium “Herbario de Plantas Útiles Efraím Hernández X” at the UACH, in the State of México, México, for future reference. Aqueous extract was obtained by using 50 g of dried and powdered leaves, in a process of infusion in 500 mL of boiling water. The aqueous extract was separated from the residues by gravity filtration; samples were frozen in liquid nitrogen and then lyophilized during 12 h using a Heto FD3 Lab lyophilizer to yield 1.11 g (2.22%). 

### 2.2. HPLC Quantification of Flavonoids

An HPLC analysis was performed using an Agilent Technologies Chromatograph. The separation was performed on an ODS Hypersil C18 column (15 mm × 4 mm i.d. and 5 *μ*m particle size). The samples were injected through a 20 *μ*L loop. The column was thermostatically controlled at 30°C using a 1 mL/min flow rate. The mobile phase consisted of 15 : 85 acetonitrile-trifluoroacetic acid solution at pH 2.5. The detection was monitored at 350 nm wavelength, and the system was run for 15 min. Calibration curves were done for the standards: kaempferol, hesperidin, naringenin, naringin, rutin, isoquercitrin, and quercetin.

### 2.3. *α*-Glucosidase Assay *In Vitro*

*α*-Glucosidase inhibition was measured at pH 7.0 and 30°C using *p*-nitrophenyl-*α*-D-glucopyranoside (pNPG) as substrate and 0.75 IU/mL of yeast *α*-glucosidase in 0.1 M sodium phosphate buffer. Acarbose and aqueous extract were dissolved in phosphate buffer, and serial dilutions from 1.4 to 0.2 mg/mL were prepared. The increments in absorption at 405 nm, due to the hydrolysis of pNPG by the *α*-glucosidase, were determined on a microplate reader DTX 880 Multimode Detector from Beckman Coulter. Ten *μ*L of acarbose or extract solution (in triplicate) were incubated during 5 min with 20 *μ*L of enzyme stock. After incubation, 10 *μ*L of substrate were added and further incubated for 35 min at 30°C. Finally, the reaction was stopped by adding 30 *μ*L of 5 M Na_2_CO_3_. The concentration required to inhibit the enzyme activity by 50% (IC_50_) was calculated by regression analysis, using the following equation:

(1) $$\begin{array}{c} v=\frac{A_{100}}{1+{\left(I/\text{I}{\text{C}}_{50}\right)}^{s}}, \end{array}$$

where *v* is the percentage of inhibition, *A*_100_ is the maximum inhibition, *I* is the inhibitor concentration, IC_50_ is the concentration required to inhibit the enzyme activity by 50%, and *s* is the cooperative degree.

### 2.4. Enzyme Kinetics

The mode of inhibition for *α*-glucosidase was determined by the Lineweaver-Burk plots. All the results are expressed as the mean of at least three experiments ± SEM. Kinetic parameters such as *V*_*m*_, *K*_*m*_, and *K*_*i*_ were evaluated by using the nonlinear regression method, based on the following inhibition equation:

(2) $$\begin{array}{c} v=\frac{v_{\max}S}{K_{m}\left(1+I/K_{i}\right)+S\left(1+I/K_{i}^{\prime }\right)}, \end{array}$$

where *v* is the initial velocity in either the Presence or absence of the inhibitor *S* and *I* are the concentrations of substrate and inhibitor, respectively; *V*_*m*_ is the maximum velocity, *K*_*m*_ is the Michaelis-Menten constant, *K*_*i*_ is the competitive inhibition constant, and *K*_*i*_′ is the uncompetitive inhibition constant. The kinetic data were analyzed using a computer program for nonlinear regressions (Origin 8.0).

### 2.5. Experimental Animals

Male Wistar normoglycemic rats, weighing 200–250 g, were obtained from our animal facilities at Facultad de Estudios Superiores Iztacala, U.N.A.M. Procedures involving animals and their care fulfilled the Mexican Official Norm for Animal Care and Handling (NOM-062-ZOO-1999) and were in compliance with international rules on care and use of laboratory animals. Furthermore, clearance for conducting the studies was obtained from the Ethics Committee for the Use of Animals in Pharmacological and Toxicological Testing of Facultad de Estudios Superiores Iztacala, U.N.A.M. For the pharmacological studies, groups of four animals were used. All doses are in mg/kg of body weight. The rats were housed in groups of four under standard laboratory conditions (12 h light/dark cycle at 22 ± 1°C) and maintained on a standard pellet diet and water *ad libitum*.

### 2.6. Preparation of the Test Samples and Determination of Glycemia

All aqueous administrations were suspended in 0.9% saline solution and given by intragastrical route. Acarbose (Sigma-Aldrich Co., St. Louis, MO, USA) was used as antihyperglycemic drug. Sucrose (Reasol, Reactivos Analíticos, México) was used as carbohydrate to carry out the sucrose tolerance tests. The control rats received only vehicle (saline solution) in the same volume (0.5 mL of vehicle/100 g of body weight) by the same route. Blood samples were collected from the caudal vein by performing a small incision at the end of the tail. Blood glucose (mg/dL) was estimated using a commercial glucometer (Accu-Chek sensor, Roche, Mannheim, Germany). The variation of glycemia as percentage for each group was calculated with respect to the initial (0 h) level, according to the following equation:

(3) $$\begin{array}{c} \%\;\text{Variation of glycemia}=\left[\frac{G_{t}-G_{i}}{G_{i}}\right]\times 100\%, \end{array}$$

where *G*_*i*_ is the initial glycemia values and *G*_*t*_ is the glycemia value after treatments administration [21].

### 2.7. Oral Sucrose Tolerance Test (OSTT)

Rats were fasted during 12 h before the experiment having free access to water. The crude extract of *A. macroprophyllata *was tested at the doses of 100, 300, and 500 mg/kg. Acarbose (5 mg/kg) was suspended in the same vehicle. Time 0 min was set before treatment with the extract; 30 min later a sucrose load (3 g/kg) was administered to the rats. Blood samples were obtained 15, 30, 60, 90, and 120 min after the carbohydrate load [21].

### 2.8. Statistical Analysis

Data represent the mean ± SEM of *n* = 4 rats for the OSTT and *n* = 3 assays for *in vitro* experiments. Differences were analyzed using Student's *t*-test (Sigma Stat version 3.0), and significant differences were considered at *P* < 0.05.

## 3. Results and Discussion

In México, it has been reported the use of *A. muricata, A. glabra,* and *A. cherimola* as antidiabetic species [18], which suggested to us that *A. macroprophyllata *is an interesting species to study, since it may have the same metabolic pathways that synthesize the compounds as the other members of the genus and thus may show their therapeutic properties. That is, we tested a chemotaxonomic criterion for the study.

**Figure 1:**
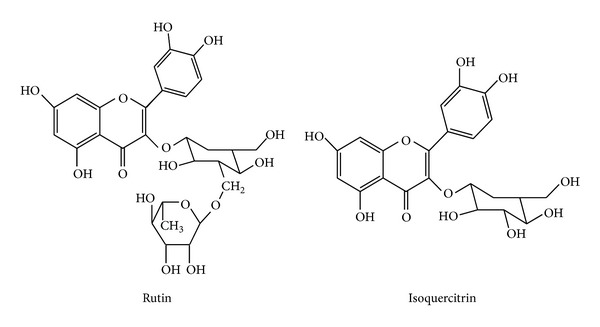
Structure of flavonoids rutin and isoquercitrin.

Elution of *A. macroprophyllata *leaves aqueous extract yielded rutin (2.88 *μ*g/mg of crude extract; RT 3.973 min) and isoquercitrin (0.71 *μ*g/mg of crude extract; RT 6.872), as well as some traces of astragalin/isoquercitrin (0.03 *μ*g/mg of crude extract) (Figure 1). 

*Annona macroprophyllata *ethanolic extract has been investigated as antinociceptive, anti-inflammatory, and anticonvulsant agents [15–17]; however, the species has not been tested for antidiabetic actions, as their counterparts have done [19, 20, 22–25]. Our study found rutin as one of its components, as well as isoquercitrin; these two flavonoids, along with quercetin, have been tested on diabetic rats, that is, rutin decreased fasting plasma glucose, increased insulin levels, and improved the antioxidant status by decreasing lipid peroxidative products, and increasing enzymic and nonenzymic antioxidants [26]. Rutin has been associated with marked decrease of hepatic and cardiac levels of triglycerides and elevated glycogen which suggest that rutin can improve hyperglycemia and dyslipidemia, while inhibiting the progression of liver and heart dysfunction in STZ-induced diabetic rats [27], and rutin has hypoglycemic effect in normal and diabetic rats [28–30]. Rutin metabolites are capable of inhibiting *α*-glucosidase activity both *in vivo* and *in vitro *[31], and the formation of advanced glycation end products, formed via protein glycation which correlates with aging and diabetes complications [32].

To our knowledge, this is the first report that the aqueous extract from *A. macroprophyllata *inhibited the activity of yeast *α*-glucosidase, with a low IC_50_ (1.18 mg/mL), very similar to that of acarbose (0.27 mg/mL). This result agrees with that reported for rutin as inhibitor of *α*-glucosidase (IC_50_ = 0.196 mM), compared with acarbose (IC_50_ 0.091 mM) [33]; therefore rutin might participate in the aqueous extract of *A. macroprophyllata *on the *α*-glucosidase inhibition and antihyperglycemic effects.

**Figure 2:**
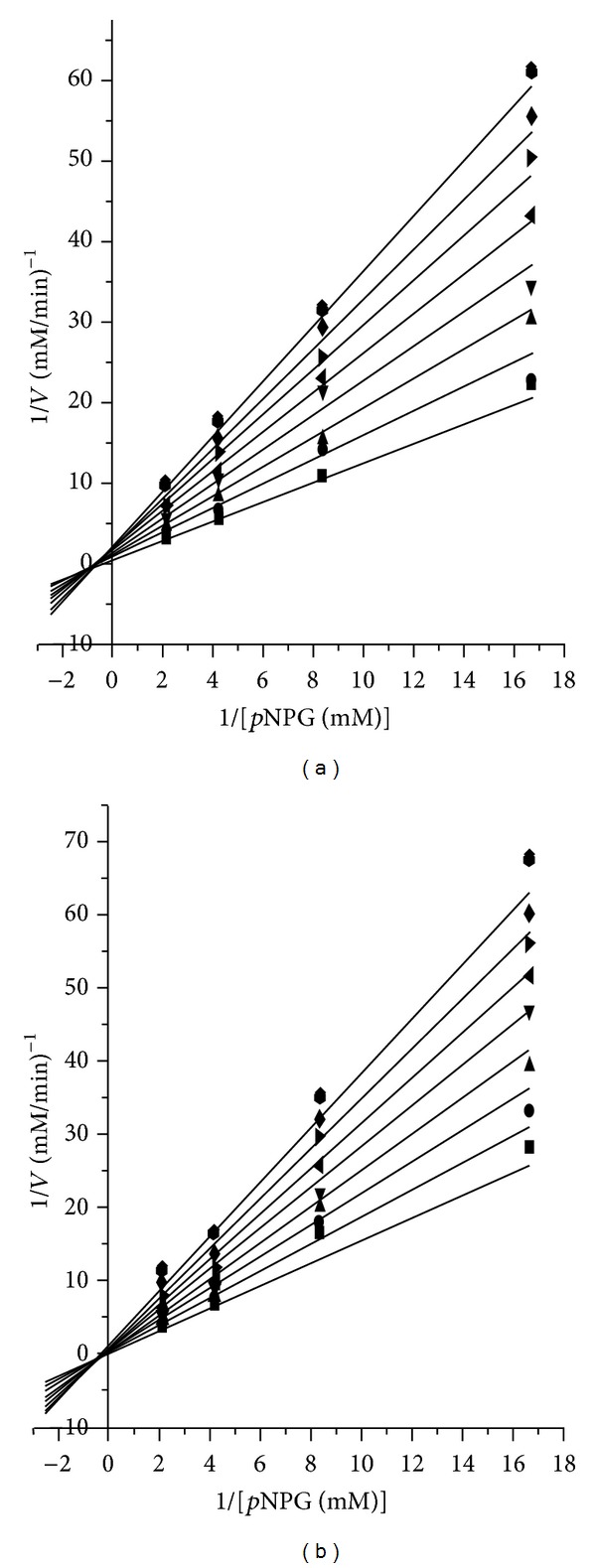
Lineweaver-Burk plots of *α*-glucosidase inhibition at different concentrations of (a) acarbose and (b) extract aqueous from *A. macroprophyllata*. 0.0 mg/mL (■), 0.2 mg/mL (*⚫*), 0.4 mg/mL (▲), 0.6 mg/mL (*▼*), 0.8 mg/mL (◂), 1.0 mg/mL (▸), 1.2 mg/mL (◆), 1.4 mg/mL (*⬢*).

The next step was to determine the nature of the inhibition exerted by the aqueous extract of *A. macroprophyllata*, then a kinetic analysis of the inhibition of enzyme activity using different amounts of aqueous extract or acarbose was conducted. The results yielded a *K*_*i*_ of 0.97 mg/mL and *K*_*i*_ of 0.79 mg/mL to aqueous extract and acarbose, respectively. These values show that the extract inhibited the enzyme and could be used to control postprandial hyperglycemia. Lineweaver-Burk plots for *α*-glucosidase from yeast, in the presence of acarbose or *A. macroprophyllata *extract at different concentrations (Figure 2), revealed typical curves for competitive inhibitors implying binding to the catalytic site. Then, we suggest that disaccharidases are targets of flavonoids in the regulation of glucose release and consequently glucose absorption by the gut. This type of inhibitor has several advantages over others; that is, its inhibition is not permanent, and undesirable effects are easily attenuated with decreasing dose; therefore, the aqueous extract of *A. macroprophyllata *represents a good alternative to avoid hyperglycemia.

**Figure 3:**
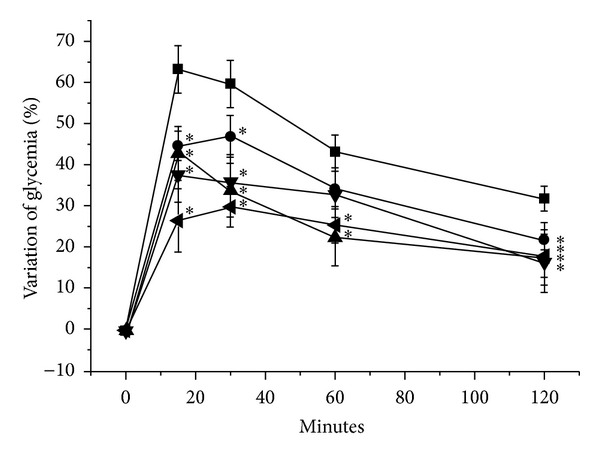
Effects of the crude extract of the leaves of *A. macroprophyllata* on blood glucose levels in rats on the OSTT. Vehicle (■), acarbose 5 mg/kg (◂), extract *A. macroprophyllata* 100 mg/kg (*⚫*), extract *A. macroprophyllata* 300 mg/kg (▲), and extract *A. macroprophyllata* 500 mg/kg (▼). Each value is the mean ± SEM for 4 rats in each group. *P < 0.05 is significantly different. *t*-test for comparison with respect to negative control values at the same time. Baseline glycemia 77 mg/dL.

On the other hand, to confirm whether our results *in vitro *could be reproduced in a whole organism, the response on glycemia after single oral sucrose ingestion was examined for *A. macroprophyllata*. As observed in Figure 3, the test extract significantly avoided the hyperglycemic response, at doses of 100, 300, and 500 mg/kg compared to control (*P* < 0.05). The postprandial blood glucose peak was diminished from 15 min in all three doses (100, 300, and 500 mg/kg); however, the highest activity was 300 > 500 > 100 mg/kg throughout the time-curve postingestion of sucrose. The glycemic lowering effect of *A. macroprophyllata *at doses of 100, 300, and 500 mg/kg was not greater than that with acarbose, a therapeutic drug used as positive control (5 mg/kg); however, maximum effect obtained at a dose of 300 mg/kg was not statistically different with acarbose dose from 60 min on, results *in vivo *that agree to the reported for other species of the genus *Annona* as is the case of *A. squamosa*, *A. muricata,* and *A. cherimola*, which also show antioxidant, hypoglycemic, antihyperglycemic, and hypolipidemic properties [19, 20, 22–25]. These data support the idea that this species synthesizes similar secondary metabolites that act as antidiabetic, directly by lowering glucose availability in the gut or indirectly by decreasing oxidative stress and/or lipids, attenuating the effect of these two factors in disease. Our results indicate that the control of postprandial glycemia showed by *A. macroprophyllata *involves an antihyperglycemic effect, mediated by the regulation of glucose uptake from the intestinal lumen, through the inhibition of carbohydrate digestion, putatively by inhibition of intestinal *α*-glucosidases complex, as observed *in vitro *for yeast *α*-glucosidase. In addition, the search for the active principle of the extract could generate a new *α*-glucosidase inhibitor, useful for the development of new antidiabetic or antiobesity agents.

In conclusion, our results indicate that *A. macroprophyllata *aqueous extract has potent antihyperglycemic effect, and according to the *in vitro* studies it acts as a competitive inhibitor of the *α*-glucosidase. Last but not least, this study provides scientific support to use *A. macroprophyllata *leaves in Mexican traditional medicine for the treatment of DM2.
